# Manual Versus Hammer Percussion Frequency-Domain Acoustic Screening for Hip Fracture: A Comparative Study

**DOI:** 10.7759/cureus.90975

**Published:** 2025-08-25

**Authors:** Etaro Hashimoto, Shoichi Masumoto, Mikiya Sato, Tetsuhiro Maeno

**Affiliations:** 1 Department of Primary Care and Medical Education, University of Tsukuba, Tsukuba, JPN; 2 Department of Family Medicine, General Practice, and Community Health, University of Tsukuba, Tsukuba, JPN; 3 Department of Health Services Research, University of Tsukuba, Tsukuba, JPN; 4 Department of Occupational Safety and Health, Human Resources Group, Sumitomo Heavy Industries, Ltd., Tokyo, JPN

**Keywords:** acoustic diagnosis, frequency analysis, hip fracture, percussion, screening

## Abstract

Introduction

Delayed diagnosis of hip fracture (HF), one of the most common fractures encountered in clinical practice, is associated with serious complications or adverse outcomes. However, these fractures are frequently missed on radiography. Auscultatory percussion, a simple screening method that compares percussion sounds between lower limbs using a stethoscope, has limitations owing to its subjective evaluation, which can lead to unstable diagnostic accuracy. Therefore, this study aimed to verify whether objective frequency analysis of percussion sounds could overcome this issue to achieve high-accuracy diagnosis. We further compared the diagnostic performance of manual percussion versus percussion with a tendon hammer.

Materials and methods

This case-control study enrolled 40 patients with HFs (fracture group) and 20 hospitalized patients without fractures (control group). All patients underwent percussion of the pubic symphysis using manual percussion and a tendon hammer, and sounds were recorded from both patellae. The absolute value of the sound pressure difference between the sides was calculated across 512 frequency bands (0-24,000 Hz). Diagnostic performance was evaluated using group comparisons (Mann-Whitney U test), receiver operating characteristic (ROC) analysis with bootstrap internal validation, decision curve analysis (DCA), and multivariate logistic regression analysis.

Results

Manual percussion demonstrated the highest diagnostic performance at the 2718.75-Hz band, with an optimism-corrected area under the curve (AUC) of 0.923 (95% confidence interval: 0.845-0.979). At a cutoff value of 1.28 dB, the sensitivity and specificity were 97.5% and 69.8%, respectively, yielding an extremely low negative likelihood ratio of 0.04. Hammer percussion also showed good performance in the 421.88-Hz band (AUC: 0.861), although the difference in diagnostic performance between the two methods was not statistically significant. DCA confirmed the clinical utility of both methods. In multivariate analysis, the sound pressure difference for both methods was a significant independent predictor of fracture. Furthermore, exploratory subgroup analysis suggested the diagnostic performance of manual percussion was stable across key patient subgroups.

Conclusion

These results indicate that objective frequency-domain analysis of percussion sounds, particularly with manual percussion, represents a simple and effective screening tool for ruling out HFs, given its high sensitivity and excellent negative likelihood ratio. This study revealed new scientific insight that the diagnostically effective frequency band depends on the physical properties of percussion. These findings overcome the challenges of conventional subjective diagnostic methods and could contribute to the future development of accessible, objective, and non-invasive fracture diagnosis technologies.

## Introduction

Hip fracture (HF) is a serious injury common in older adults, particularly those with established risk factors, such as female sex, osteoporosis, and low body mass index (BMI) [1,2]. Due to the global demographic trend of an aging population, the incidence of HF is increasing, with current estimates projecting that cases will exceed six million annually by 2050 [3]. Further, one-year mortality after HF ranges from 24% to 28%, depending on fracture type, representing a substantial healthcare burden [4].

HF is typically diagnosed as an initial step by radiography. However, this approach has considerable limitations, as diagnosis is delayed in 2-9% of cases owing to subtle symptoms or unclear fracture lines [5]. These delays can lead to unnecessary patient suffering, increased risk of complications, and potentially more invasive surgeries. One meta-analysis reported that 39% of HFs were initially missed on radiographs [6]. Although magnetic resonance imaging (MRI) is the gold standard, its availability is limited, especially in resource-constrained settings [7]. Furthermore, in settings such as nursing homes where radiography examinations are unavailable, diagnostic delays can necessitate more invasive surgeries and increase the risk of complications. This is a particular challenge in developing countries where imaging resources may be scarce [8]. To mitigate this, an objective, simple, and non-invasive screening tool with high sensitivity capable of reliably ruling out fractures is needed that is usable not only by specialists but also by non-experts (including paramedics and caregivers) and in diverse settings.

While auscultatory percussion, i.e., the patellar-pubic percussion test (PPPT), is a screening method in which percussion sounds are evaluated using a stethoscope [9], it has substantial limitations, including low interobserver agreement due to its subjective nature, difficulty in applying consistent percussion force, and the fact that percussion is a technique that requires proficiency to conduct [10,11]. The availability of a simple procedure performed by non-experts would enable a wider range of personnel, e.g., nurses, paramedics, and caregivers, to participate in fracture screening. This highlights the need for a novel acoustic diagnostic method that is not only based on objective indicators but is also sufficiently simple to be used by non-experts.

Recent advances in acoustic analysis technology enable objective evaluation to overcome the subjectivity of the PPPT, with studies combining tuning forks and electronic stethoscopes showing potential [12,13]. However, these prior attempts at objective acoustic diagnosis have focused on sound amplitude (loudness). One key report achieved a sensitivity of only 78% and a specificity of 82%, which is insufficient for a reliable screening tool. Crucially, these studies have largely overlooked the role of frequency (pitch) [12]. Physically, high-frequency sound waves are markedly affected by obstacles [14]. This led us to hypothesize that bone discontinuity from a fracture would cause more prominent changes in specific frequency bands, detectable via techniques like fast Fourier transform (FFT), thereby markedly improving diagnostic accuracy. The critical research gap is that no prior studies have systematically investigated which specific frequency bands are most diagnostically valuable or how different percussion methods affect these signals.

To address these knowledge gaps, the primary aim of this study was to determine if an objective, frequency-domain analysis of percussion sounds could achieve the high sensitivity required for an effective HF screening (rule-out) tool. Furthermore, the optimal percussion method for non-expert use remains unknown. Therefore, the secondary aim was to compare the diagnostic performance of traditional manual percussion, a technique that requires proficiency to conduct, with percussion using a common tendon hammer, a mechanically simpler approach, to assess the optimal method for non-expert use.

A preliminary version of the concept for this study, using a different protocol and dataset, was previously presented (poster: Hashimoto E, Ozone S, Haruta J, Goto R, Kinoshita K. Visualization of Auscultatory Percussion with a Smartphone to Diagnose Proximal Femoral Fractures. 48th Annual Meeting of the North American Primary Care Research Group (NAPCRG); November 20-24, 2020).

## Materials and methods

Study design

This case-control study was approved by the Ethics Committee of the University of Tsukuba (approval number: 2037). All procedures followed the principles of the Declaration of Helsinki, and written informed consent was obtained from all participants.

Participants

For the fracture group, we enrolled a consecutive cohort of all patients admitted to Mito Kyodo General Hospital, an affiliated hospital of the University of Tsukuba, for a new HF between October 2024 and March 2025 who met the eligibility criteria. This systematic approach minimized selection bias for the case group. The control group was recruited via convenience sampling from the same hospital during the same period. Our rationale was that matching for age (≥75 years) and hospitalization status would inherently create a control group with a baseline health status, including a similar burden of comorbidities and frailty, comparable to that of the fracture group. Therefore, we enrolled patients from the general internal medicine and post-acute care wards. This strategy was considered more appropriate than recruiting healthy community dwellers, as it is crucial for mitigating spectrum bias and ensuring that the performance of the test is evaluated in a clinically relevant population. While the use of convenience sampling for the control group is an acknowledged limitation, this pragmatic approach was deemed essential for constructing a clinically comparable cohort. The age criteria were based on Japanese epidemiological data showing a sharp increase in the incidence of HF after 75 years of age [15].

Exclusion criteria included bilateral fractures, concomitant pelvic or patellar fractures, history of relevant fractures or surgery, limb defects, acute lower limb conditions other than fractures, non-fracture-related pain, or inability to obtain consent.

Procedures

The reference standard was the final diagnosis of HF confirmed by radiographic imaging (radiography, computed tomography (CT), or MRI) and reviewed by an orthopedic surgeon.

Data were collected by eight trained examiners. The examiners were not blinded to the patient groups; however, bias was minimized by using an objective, algorithm-based acoustic analysis, as described later. A definitive diagnosis of HF was established prior to acoustic measurements. Therefore, the acoustic measurement results did not influence the final diagnostic decision. To enhance reproducibility, the percussion force was standardized to 200-250 g based on preliminary measurements and training with a pressure sensor (RP-C-MK01X, Shenzhen LEGACT Technology Co., Ltd., Shenzhen, China).

During percussion, participants were placed in the supine position, and measurements were conducted at the patient's bedside. To minimize environmental noise, efforts were made to ensure the surroundings were as quiet as possible; in multi-bed rooms, privacy curtains were drawn to create a partitioned space. Care was taken to adjust the positions of the participant's lower limbs to be as symmetrical as possible. In patients with HF, if the affected limb was externally rotated, the contralateral limb was adjusted to a similar degree of rotation. Following this positioning, each participant's pubic symphysis was percussed 30 times with manual percussion and with a Kudo-type tendon hammer (Yufu Itonaga Co., Ltd., Tokyo, Japan). This setup, which evaluates the sound conducted from a single source to both lower limbs, was designed to minimize the variability compared with the traditional PPPT method.

**Figure 1:**
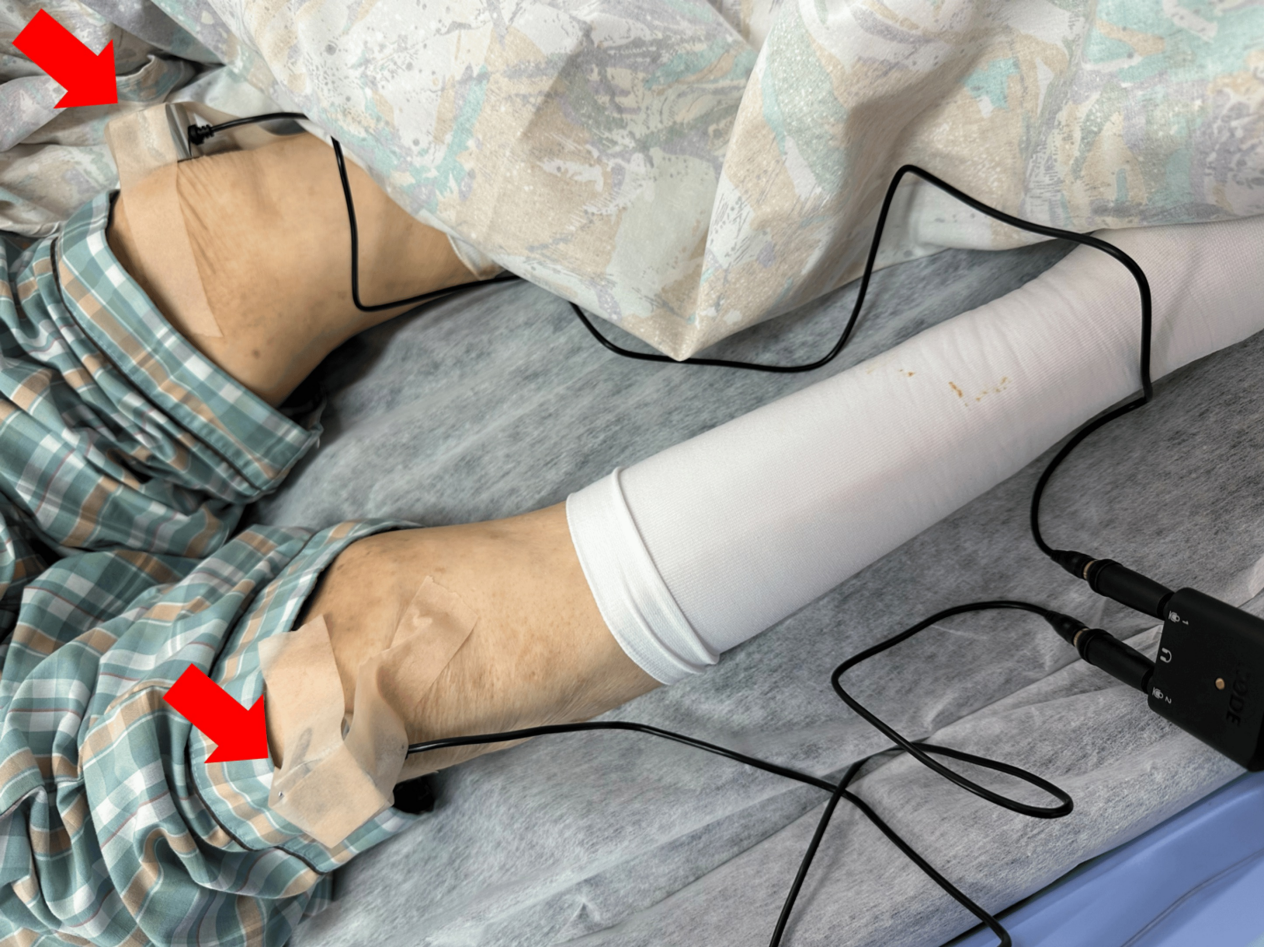
Experimental setup for acoustic data acquisition. This image illustrates the measurement procedure in a representative patient with a hip fracture. The core principle of this method is to compare the sound transmission characteristics between the fractured and unfractured sides. To achieve this, the pubic symphysis is percussed, and the conducted sound is recorded by small condenser microphones (red arrows) placed on each patella. To ensure measurement symmetry, the unaffected limb is positioned to match the external rotation of the fractured limb.

The sounds were recorded at the center of each patella using small condenser microphones (MIC-FL330-SET2, Sun-Mechatronics, Tokyo, Japan). The microphones were affixed to the participant's skin using medical tape with gentle, uniform pressure to ensure good acoustic contact without causing discomfort. The microphones were connected to a smartphone (iPhone 15, Apple Inc., Cupertino, California, United States) running the RØDE Reporter application (Freedman Electronics Pty Ltd., Sydney, Australia) (Figure 1; Videos 1-2).

**Video 1:** Manual percussion of the pubic symphysis using a mannequin.

**Video 2:** Hammer percussion of the pubic symphysis using a mannequin.

Using the default settings of the application, the audio was recorded in an uncompressed WAV format at a sampling rate of 48 kHz and a bit depth of 24 bits.

For all participants, index tests were performed during hospital admission. In the fracture group, this was typically within 24 hours of the reference standard diagnosis. No adverse events related to the percussion method were observed. While formal pain scores were not systematically collected, no participants reported prohibitive pain during the procedure.

Acoustic analysis and data processing

The recorded audio was analyzed using acoustic analysis software (Adobe Audition v25.3, Adobe Inc., San Jose, California, United States). The selected recording parameters (sampling rate: 48 kHz) ensured a Nyquist frequency, defined as half the sampling rate (24 kHz), which covers the entire frequency range of interest. The internal audio interface of the smartphone incorporates standard anti-aliasing filters to attenuate frequency components above the Nyquist frequency and thus prevent aliasing. No additional digital filtering was applied after recording. The sound pressure level was expressed in decibels relative to full scale (dBFS), where 0 dBFS corresponds to the maximum possible digital amplitude and lower levels are expressed as negative values.

**Figure 2:**
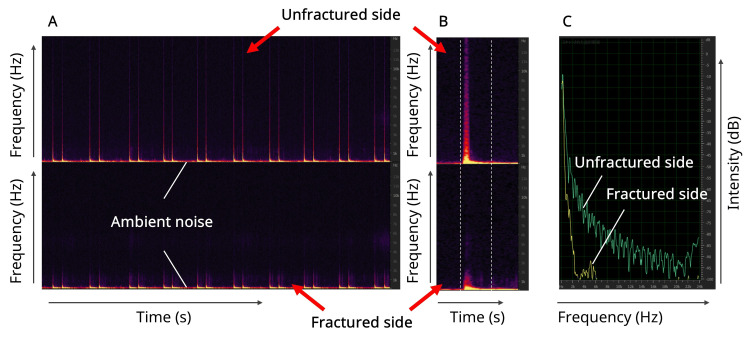
Overview of the acoustic analysis process. (A) Spectrograms of conducted percussion sounds from a representative case, showing the unfractured (top panel) and fractured (bottom panel) sides. The horizontal axis presents time (s), vertical axis presents frequency (Hz), and color brightness indicates the sound pressure intensity. Note the visible reduction in high-frequency components on the fractured side. (B) A magnified view of a single percussion sound (impulse) selected for analysis. The range for analysis was defined as the segment from which the sound emerges from ambient noise until it returns to the baseline. (C) The frequency spectrum obtained by applying a fast Fourier transform (FFT) to the single waveform selected in panel B. The horizontal axis shows the frequency (Hz), and the vertical axis shows the sound pressure level (dB). The spectra for the unfractured side (green line) and fractured side (yellow line) are overlaid. In this study, sound pressure data for 512 frequency bands were extracted from this spectrum.

From the 30 percussion sounds recorded for each method, the first 20 analyzable waveforms were selected for analysis based on a pre-defined algorithm (Figure 2A).

Waveforms contaminated by obvious external noise, e.g., conversation or doors closing, were discarded, and the subsequent waveform was considered for inclusion. This standardized selection process minimized selection bias.

For each selected waveform, the segment from sound onset to its return to the ambient noise baseline was isolated (Figure 2B), and a FFT, a mathematical method for deconstructing a complex sound into its component frequencies, was applied to generate a frequency spectrum (Figure 2C). A Blackman-Harris window was used to reduce spectral leakage during the FFT calculation. The sound pressure data were extracted for 512 frequency bands from this spectrum. To minimize the influence of outliers, the median of the 20 data points for each band was used as the final representative value for that patient.

A formal acoustic calibration of the measurement system was not performed, and the manufacturer did not provide detailed frequency response data for the microphones. However, the influence of the absolute frequency response characteristics of the microphone on the results is considered minimal.

Outcomes

The primary outcome was the absolute value of the sound pressure difference between the unaffected and affected sides of each frequency band. The absolute value was chosen to capture the magnitude of the difference regardless of the direction. Secondary outcomes included the p-value for group comparisons, area under the curve (AUC) for diagnosis, and sensitivity, specificity, and optimal cutoff value in the most effective frequency band. Across all diagnostic analyses, the primary performance values reported are the optimism‑corrected area under the ROC curve (AUC), sensitivity, and specificity with 95% confidence intervals obtained via bootstrap internal validation (2,000 resamples), unless otherwise specified.

Statistical analysis

Statistical analyses were performed using IBM SPSS Statistics for Windows (Version 30.0, IBM Corp., Armonk, New York, United States) and R (Version 4.5.1) with RStudio 2025.05.1 (Posit PBC, Boston, Massachusetts, United States). The comparison of participant characteristics and the primary multivariable logistic regression analyses were conducted using SPSS. Subsequent analyses to assess statistical robustness and clinical utility, e.g., the bootstrap internal validation, decision curve analysis (DCA), and comparison of AUCs with the DeLong test, were performed using R.

The sample size was determined a priori based on a pilot study that focused on sound pressure amplitude. This initial calculation indicated that a minimum of 18 participants per group was required to detect a 5-dB difference between groups with 90% power and a 5% significance level. As the current frequency-domain analysis was hypothesized to have diagnostic performance superior to that of an amplitude-based approach, this sample size was considered sufficient. To enhance the statistical power, all eligible patients were enrolled during the study period. To further ensure the adequacy of our sample, a post hoc assessment was performed to confirm that the achieved sample size provided sufficient precision for the primary diagnostic accuracy estimates. The estimated sensitivity of 97.5% obtained in this study had a 95% confidence interval of approximately 8 percentage points, ensuring sufficient accuracy for use as a screening test. The limited number of events per variable was acknowledged as a potential limitation affecting the stability of the multivariable analysis.

Participant characteristics were compared using the Student t-test for normally distributed continuous variables, the Mann-Whitney U test for non-normally distributed continuous variables, and the Fisher exact test for categorical variables. The Shapiro-Wilk test was used to assess normality. For the primary outcome, the non-parametric Mann-Whitney U test was used to compare the sound pressure difference between the fracture and control groups for each of the 512 frequency bands spanning 0-24 kHz. The Benjamini-Hochberg procedure was used to control the false discovery rate (FDR) (target q=0.05) for these multiple comparisons. Among bands that remained significant after FDR control, the optimal frequency band was defined as the one with the highest AUC. For screening purposes, the diagnostic cutoff was chosen to prioritize high sensitivity (≥90%) rather than maximizing the Youden index.

To address the risk of overfitting and performance overestimation from selecting the optimal frequency and cutoff within the same dataset, we performed bootstrap internal validation with 2,000 resamples to estimate optimism in the apparent performance metrics (AUC, sensitivity, and specificity). The final reported performance metrics are the optimism‑corrected values with 95% confidence intervals derived from the bootstrap distribution. This study was a discovery analysis at the individual‑bin level. We did not prespecify frequency band families or perform nested cross‑validation; instead, we controlled the FDR and applied bootstrap optimism‑correction for apparent performance.

To assess clinical utility, we calculated positive and negative likelihood ratios (LR+ and LR−) and conducted DCA to evaluate net benefit across clinically plausible threshold probabilities, in comparison with treat‑all and treat‑none strategies.

We compared AUCs for manual versus hammer percussion using the DeLong test; optimism‑corrected performance metrics are reported as the primary results. To assess robustness, we conducted a sensitivity analysis by repeating the primary analyses after excluding patients with a diagnosis of hip or knee osteoarthritis (OA).

To evaluate the consistency of the diagnostic performance, exploratory subgroup analyses were performed. Specifically, we assessed whether the AUC of the acoustic measurement test differed across subgroups stratified by fracture type and major factors identified in prior research as being clinically associated with HF. Known HF risk factors based on the literature include age (stratified by the median), sex, osteoporosis, and BMI. The BMI cutoff value was set at 22 kg/m² in accordance with a previous study [2]. To formally test for performance differences, a logistic regression model with an interaction term was constructed.

Furthermore, multivariate logistic regression analysis was performed to assess the independent diagnostic ability of the acoustic measurements after adjusting for potential confounders.

Reporting guideline

This study was conducted in accordance with the Standards for Reporting of Diagnostic Accuracy Studies (STARD) 2015 guidelines [16].

## Results

Breakdown of the analyzed participants

**Figure 3:**
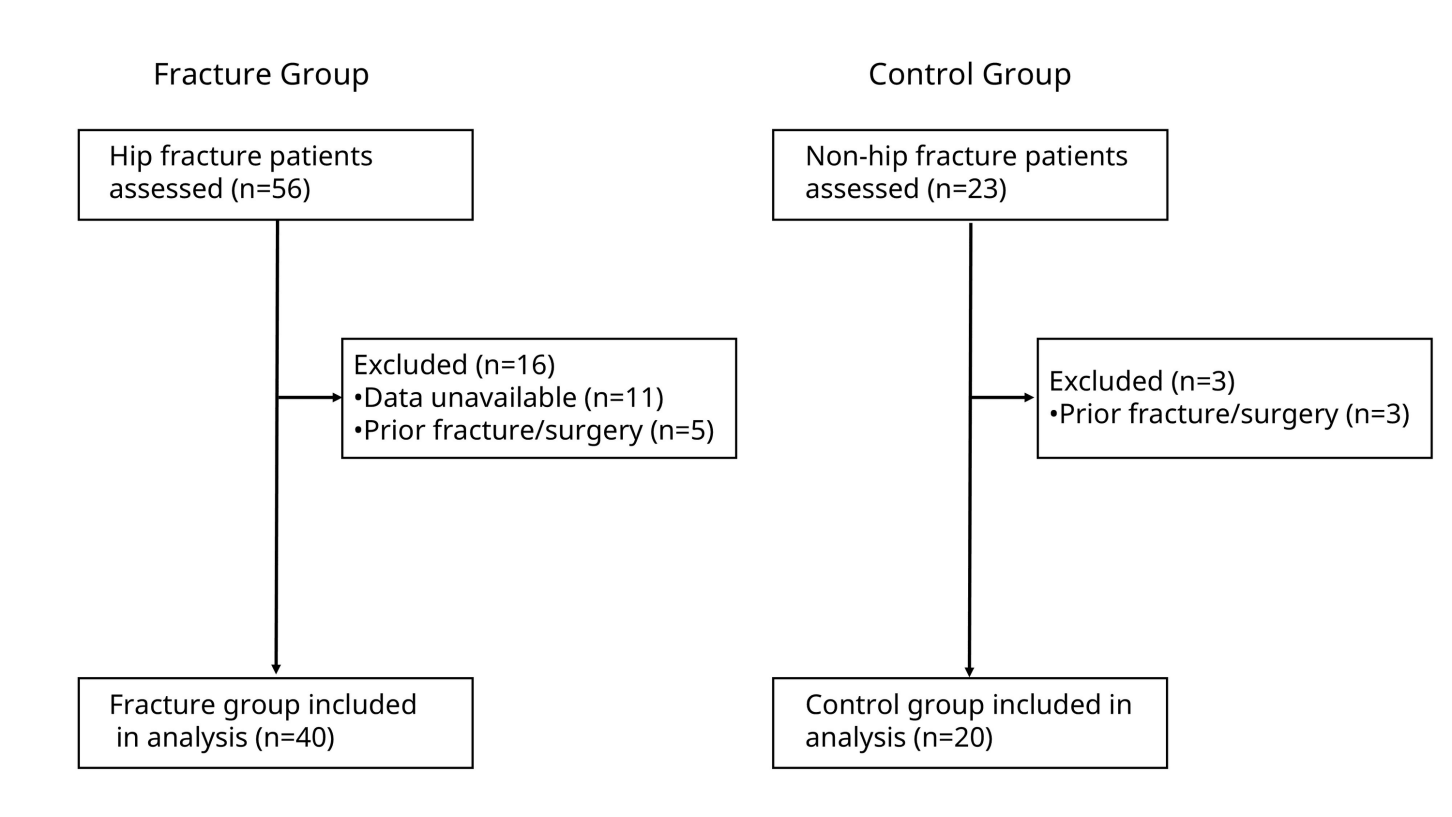
Flow diagram of participant selection. This diagram shows the process of participant enrollment and exclusion for the fracture and control groups. A total of 56 patients with hip fractures and 23 patients without hip fractures were initially assessed for eligibility. After excluding patients on the basis of predefined criteria, a final sample of 40 patients in the fracture group and 20 patients in the control group was included in the analysis.

A flow diagram of participant selection is shown in Figure 3. No indeterminate results were encountered in the acoustic analysis.

Participant characteristics

The baseline characteristics of the enrolled participants are shown in Table 1.

**Table 1:** Baseline characteristics of the study participants. Data are presented as mean±standard deviation or as median (interquartile range (IQR)) indicated by an asterisk (*). Statistical comparisons were made using the following: ^a^ Student t-test, ^b^ Fisher exact test, or ^c^ Mann-Whitney U test. P-values <0.05 were considered statistically significant.

Characteristic	Fracture group (n=40)	Control group (n=20)	P-value
Age (years)	82.5±4.7	79.1±4.5	0.049^a^
Male (n (%))	10 (25)	7 (35)	0.545^b^
Female (n (%))	30 (75)	13 (65)	
Height (cm)*	154.1 (150.2-156.2)	155 (152.3-158.3)	0.642^c^
Weight (kg)*	50.3 (46-54.9)	51.2 (45.7-55.2)	0.587^c^
Body mass index	20.7±4.2	21.6±4.0	0.467^a^
Leg length (cm)	80.7±5.9	83.0±5.1	0.154^a^
Thigh length (cm)*	40.1 (38.5-42.6)	41.2 (39-43.2)	0.583^c^
Osteoporosis (n (%))	11 (27.5)	4 (20)	0.531^c^
Hip osteoarthritis (n (%))	0 (0)	1 (5)	0.157^c^
Knee osteoarthritis (n (%))	2 (5)	3 (15)	0.190^c^
Side of fracture			
Right (n (%))	27 (67.5)	-	-
Left (n (%))	13 (32.5)	-	-
Type of fracture			
Femoral neck fracture (n (%))	24 (60)	-	-
Trochanteric fracture (n (%))	16 (40)	-	-

The fracture group was significantly older than the control group (82.5±4.7 versus 79.1±4.5 years; p=0.049). No significant differences were found in other demographic variables, including sex, BMI, or the prevalence of osteoporosis or OA. For patients in the fracture group, the leg and thigh lengths were measured on the unfractured side. All patients with a known diagnosis of osteoporosis underwent pharmacological treatment. Within the fracture group, 67.5% of the fractures were on the right side, and 60% were femoral neck fractures.

Overall diagnostic performance and clinical utility

To address the potential for overfitting due to the data-driven selection of the optimal frequency and cutoff from 512 bands, we performed internal validation using a bootstrap procedure (2,000 resamples). The analysis revealed minimal optimism (≤0.002 for all metrics), indicating the robustness of the performance estimates. The optimism-corrected diagnostic performance metrics are summarized in Table 2.

**Table 2:** Summary of optimism-corrected diagnostic performance and clinical utility metrics. Metrics (AUC, sensitivity, and specificity) are optimism-corrected values derived from bootstrap internal validation (2,000 resamples), confirming minimal optimism (≤0.002). 95% CIs were calculated from the bootstrap distribution. AUC: area under the curve

Metric	Manual percussion (2718.75 Hz)	Hammer percussion (421.88 Hz)
Diagnostic accuracy		
AUC (95% CI)	0.923 (0.845-0.979)	0.861 (0.759-0.943)
Optimal cutoff value	1.28 dB	2.32 dB
Sensitivity (% (95% CI))	97.5 (92.3-100.0)	90 (79.1-97.8)
Specificity (% (95% CI))	69.8 (50.0-90.0)	64.9 (43.8-85.7)
Clinical utility		
Positive likelihood ratio (LR+)	3.23	2.56
Negative likelihood ratio (LR-)	0.04	0.15

Manual percussion demonstrated excellent diagnostic accuracy (AUC: 0.923). Notably, it achieved a very high sensitivity (97.5%) and an extremely low negative likelihood ratio (LR-: 0.04), indicating strong utility as a rule-out screening tool. Hammer percussion also showed good diagnostic accuracy (AUC: 0.861). However, a formal comparison using the DeLong test indicated that the difference in AUCs between the two methods was not statistically significant (p=0.235).

Clinical utility and decision curve analysis

The clinical utility is further highlighted by the likelihood ratios. Given the very low LR- of manual percussion (0.04), a negative test result substantially reduces the probability of fracture. For example, if the pre-test probability of HF was estimated at 40%, a negative result with manual percussion would reduce the post-test probability to just 2.3%.

**Figure 4:**
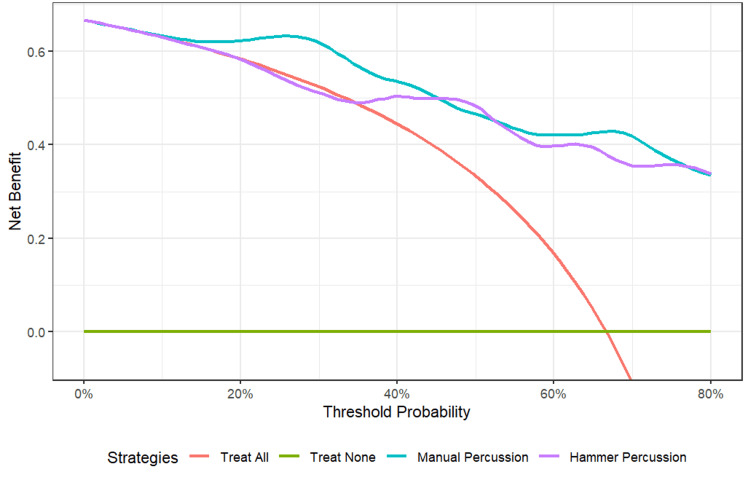
Decision curve analysis (DCA) for manual and hammer percussion. This graph illustrates the net benefit of different diagnostic strategies across a range of threshold probabilities. Both acoustic methods offer a higher net benefit than the default strategies ("Treat All" and "Treat None") over a wide range of clinically relevant probabilities (approximately 5-80%). Manual percussion consistently provides a superior or equivalent net benefit compared with hammer percussion.

DCA was performed to evaluate the net clinical benefit of the acoustic tests (Figure 4).

The analysis revealed that both methods provide a positive net benefit compared with the default strategies across a wide range of threshold probabilities (approximately 5-80%). Manual percussion consistently provided a superior or equivalent net benefit compared with hammer percussion.

Frequency-domain analysis

**Figure 5:**
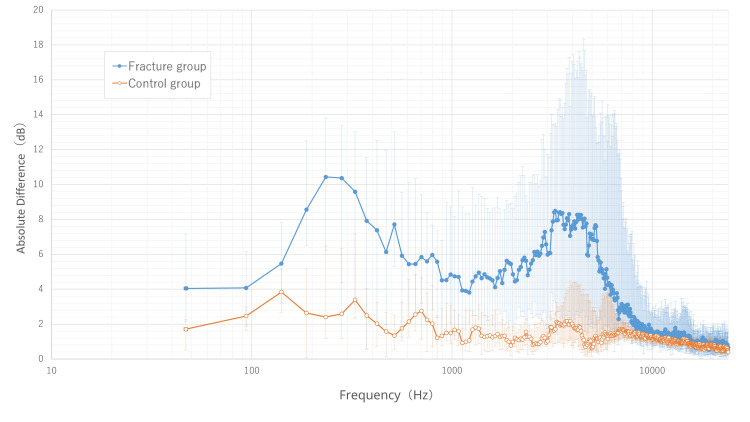
Absolute sound pressure difference across different frequency bands in manual percussion. The median values of the absolute sound pressure difference for each frequency band are plotted for the fracture (blue line, ●) and control (orange line, ○) groups. The horizontal axis shows frequency in Hertz (Hz) on a logarithmic (log_10_) scale. The error bars represent the interquartile range, with the upper and lower ends corresponding to the 75th and 25th percentiles, respectively. The graph visually demonstrates that the fracture group exhibited a markedly larger sound pressure difference than the control group across most frequencies, with this difference being most prominent in the high-frequency range of approximately 2000-5000 Hz.

In manual percussion, the fracture group showed a markedly larger sound pressure difference than the control group, particularly in the high-frequency range (2000-5000 Hz) (Figure 5).

These differences were statistically robust, remaining significant even after Benjamini-Hochberg correction (FDR <0.05).

**Figure 6:**
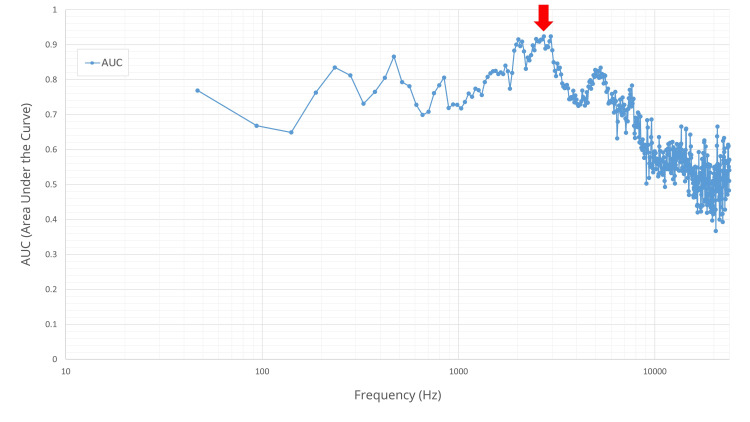
Diagnostic performance (AUC) across frequency bands for manual percussion. The AUC for fracture diagnosis is plotted for each of the 512 frequency bands. The x‑axis is frequency (Hz) plotted on a logarithmic (log_10_) scale, and the vertical axis represents the AUC value. Each blue circle marker corresponds to the AUC calculated from the absolute sound pressure difference at that specific frequency band. The plot shows that diagnostic performance varies by frequency, peaking at 2718.75 Hz (red arrow), which yielded a maximum apparent AUC of 0.924. AUC: area under the curve

ROC analysis identified the 2718.75 Hz band as optimal for diagnosis (Figure 6). At this frequency, the median sound pressure differences were 5.93 dB and 0.87 dB in the fracture and control groups, respectively (p<0.001).

**Figure 7:**
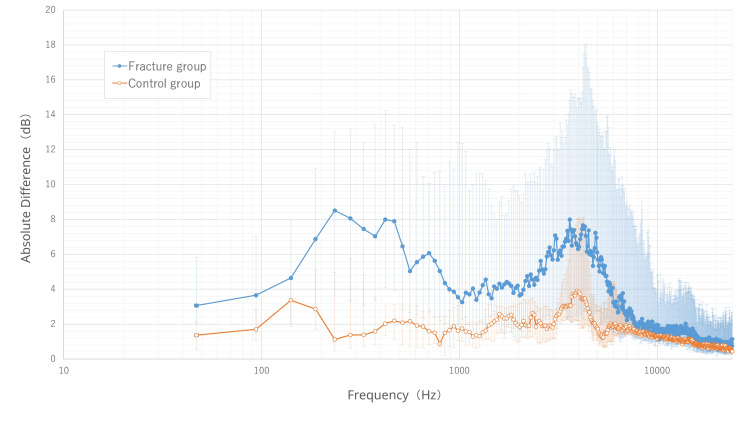
Absolute sound pressure difference across frequency bands in hammer percussion. The median values of the absolute sound pressure difference for each frequency band are plotted for the fracture (blue line, ●) and control (orange line, ○) groups. The horizontal axis shows frequency (Hz) on a logarithmic (log_10_) scale. The error bars represent the interquartile range, with the upper and lower ends corresponding to the 75th and 25th percentiles, respectively. This graph visually demonstrates that the fracture group also exhibited a larger sound pressure difference than the control group, with this difference being most prominent in the low-frequency region below 500 Hz.

For hammer percussion, the most prominent difference was observed in the low-frequency region below 500 Hz (Figure 7).

**Figure 8:**
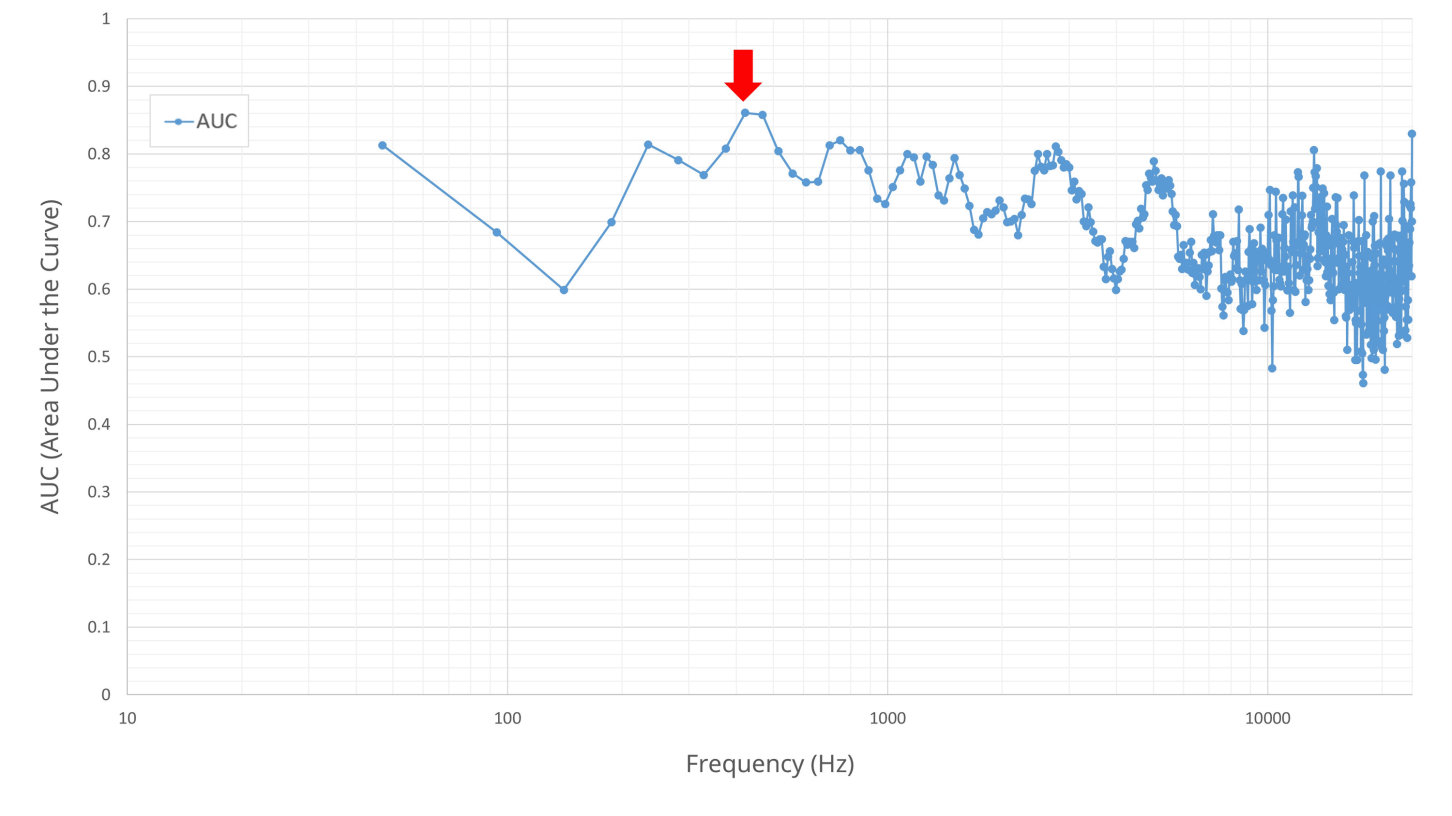
Diagnostic performance (AUC) across frequency bands in hammer percussion. The AUC for fracture diagnosis is plotted for each of the 512 frequency bands. Frequency (Hz) on the x‑axis is shown on a logarithmic (log_10_) scale. The horizontal axis represents frequency in Hertz (Hz), and the vertical axis represents the AUC value. Each blue circle marker corresponds to the AUC calculated from the absolute sound pressure difference at that specific frequency band. The plot shows that for hammer percussion, diagnostic performance was highest in the low-frequency range, peaking at 421.88 Hz (red arrow) with a maximum apparent AUC of 0.861, indicating good diagnostic accuracy. AUC: area under the curve

The optimal diagnostic frequency for this method was 421.88 Hz (Figure 8). At this frequency, the median sound pressure differences were 7.99 dB and 2.03 dB in the fracture and control groups, respectively (p<0.001).

Robustness and subgroup analyses

The stability of the diagnostic performance across various subgroups was evaluated along with comparisons between the two methods using the DeLong test (Table 3).

**Table 3:** Subgroup analysis of diagnostic performance (AUC) with method comparison and interaction testing. This table shows the diagnostic performance (AUC) of both percussion methods across various patient subgroups. The 95% CIs were calculated using the bootstrap method for robustness. ^1^p-value (DeLong test): This is the p-value from a direct comparison between the AUCs of manual and hammer percussion. A value <0.05 indicates a statistically significant difference in diagnostic accuracy between the two methods. ^2^p-value for interaction: Calculated from a logistic regression model including an interaction term, this tests whether there is a significant difference in diagnostic accuracy between the levels of a characteristic (e.g., male versus female). A value <0.05 suggests that the characteristic influences the diagnostic performance. ^3^N/A: Not applicable, indicates that the statistical test could not be performed due to the data structure. AUC: area under the curve; BMI: body mass index

Subgroup	n (fracture/control)	Manual percussion (2718.75 Hz) AUC (95% CI)	Hammer percussion (421.88 Hz) AUC (95% CI)	P-value (DeLong test)^1^	P-value for interaction^2^	
					Manual	Hammer
Overall	40/20	0.923 (0.845-0.979)	0.861 (0.759-0.943)	0.235	-	-
Fracture type						
Femoral neck fracture	24/20	0.902 (0.804-0.975)	0.804 (0.671-0.921)	0.189	N/A^3^	N/A^3^
Trochanteric fracture	16/20	0.956 (0.884-1.000)	0.947 (0.869-0.994)	0.824		
Sex						
Male	10/7	0.928 (0.846-0.981)	0.772 (0.343-0.900)	0.256	0.746	0.071
Female	30/13	0.949 (0.872-1.000)	0.921 (0.833-0.990)	0.610		
Age						
<84 years	17/9	0.902 (0.765-0.994)	0.797 (0.614-0.948)	0.213	0.488	0.155
≥84 years	23/11	0.937 (0.838-1.000)	0.921 (0.818-0.996)	0.807		
Osteoporosis						
No	29/16	0.914 (0.821-0.981)	0.834 (0.705-0.940)	0.247	0.987	0.319
Yes	11/4	0.932 (0.727-1.000)	0.955 (0.818-1.000)	0.746		
BMI						
<22 kg/m²	25/13	0.926 (0.831-0.988)	0.868 (0.742-0.969)	0.405	0.312	0.610
≥22 kg/m²	15/7	0.943 (0.829-1.000)	0.829 (0.628-0.971)	0.189		

For manual percussion, the performance was consistently high across all subgroups (all AUCs >0.90). Formal interaction testing confirmed this stability, with no significant interactions identified for any variable (all p>0.3).

In contrast, hammer percussion showed a trend toward varying performance across subgroups. Notably, the AUC was lower in males (0.772) than in females (0.921), although this interaction did not reach statistical significance (p=0.071). The DeLong test revealed no statistically significant differences between manual and hammer percussion within any subgroup (all p>0.1).

A sensitivity analysis was conducted by excluding patients with hip or knee OA (n=6) to assess the robustness of the findings (Table 4). The diagnostic performance remained robust after exclusion.

**Table 4:** Sensitivity analysis excluding patients with OA. This analysis assesses the robustness of the findings by excluding patients diagnosed with hip or knee OA (n=6). The AUC, sensitivity, and specificity were determined by ROC analysis. The 95% CIs for all metrics were calculated using the bootstrap method with 2,000 replications. P-values <0.05 were considered statistically significant. OA: osteoarthritis; AUC: area under the curve; ROC: receiver operating characteristic

Metric	Full analysis (n=60)	Sensitivity analysis (OA excluded; n=54)
Manual percussion		
AUC (95% CI)	0.923 (0.845-0.979)	0.900 (0.816-0.983)
Sensitivity (95% CI)	0.975 (0.923-1.000)	0.974 (0.921-1.000)
Specificity (95% CI)	0.698 (0.500-0.900)	0.625 (0.375-0.812)
Hammer percussion		
AUC (95% CI)	0.861 (0.759-0.943)	0.877 (0.784-0.969)
Sensitivity (95% CI)	0.900 (0.791-0.978)	0.895 (0.789-0.974)
Specificity (95% CI)	0.649 (0.438-0.857)	0.688 (0.438-0.938)
DeLong test p-value	0.235	0.677

Multivariable analysis

Multivariate logistic regression analysis was performed to assess the independent diagnostic ability of the acoustic measurements, adjusting for prespecified confounders (Table 5).

**Table 5:** Multivariate logistic regression models for predicting hip fracture. These models assess the independent diagnostic ability of the acoustic measurements from each percussion method after adjusting for potential clinical confounders (age, sex, BMI, and osteoporosis). Covariates for adjustment were prespecified and selected on the basis of two criteria: baseline characteristics showing a significant difference between groups (i.e., age) and established clinical risk factors for hip fracture (age, sex, BMI, and osteoporosis). P-values for each variable were obtained from multivariate logistic regression analysis; those <0.05 were deemed statistically significant. BMI: body mass index

Model and variable	Adjusted odds ratio	95% CI	P-value
Manual percussion			
Acoustic measurement (manual)	4.44	1.54-12.80	0.006
Age (per year)	1.07	0.90-1.26	0.447
Sex (female versus male)	0.52	0.06-4.44	0.546
BMI (per kg/m²)	1.03	0.87-1.23	0.732
Osteoporosis (yes versus no)	1.08	0.13-9.06	0.947
Hammer percussion			
Acoustic measurement (hammer)	1.62	1.18-2.22	0.003
Age (per year)	1.08	0.96-1.22	0.220
Sex (female versus male)	0.90	0.16-5.21	0.910
BMI (per kg/m²)	0.99	0.87-1.13	0.871
Osteoporosis (yes versus no)	0.62	0.11-3.63	0.596

In both models, acoustic measurements remained statistically significant independent predictors of fractures. The adjusted odds ratio (aOR) for manual percussion was 4.44 (95% CI: 1.54-12.80; p=0.006), which was substantially higher than that for hammer percussion (aOR=1.62; 95% CI: 1.18-2.22; p=0.003).

**Figure 9:**
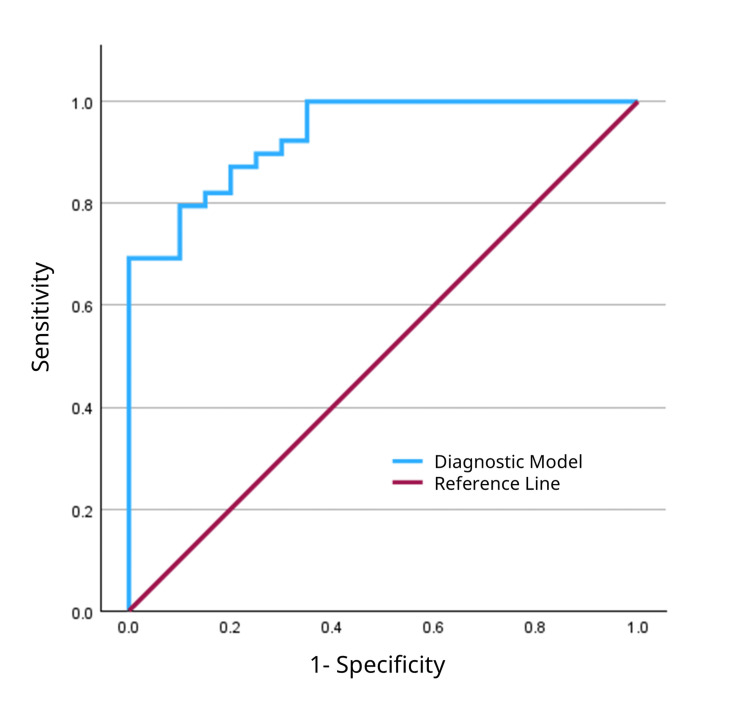
Receiver operating characteristic curve for the multivariable diagnostic model of manual percussion. The model, which was adjusted for age, sex, body mass index, and osteoporosis, achieved an area under the curve of 0.935.

**Figure 10:**
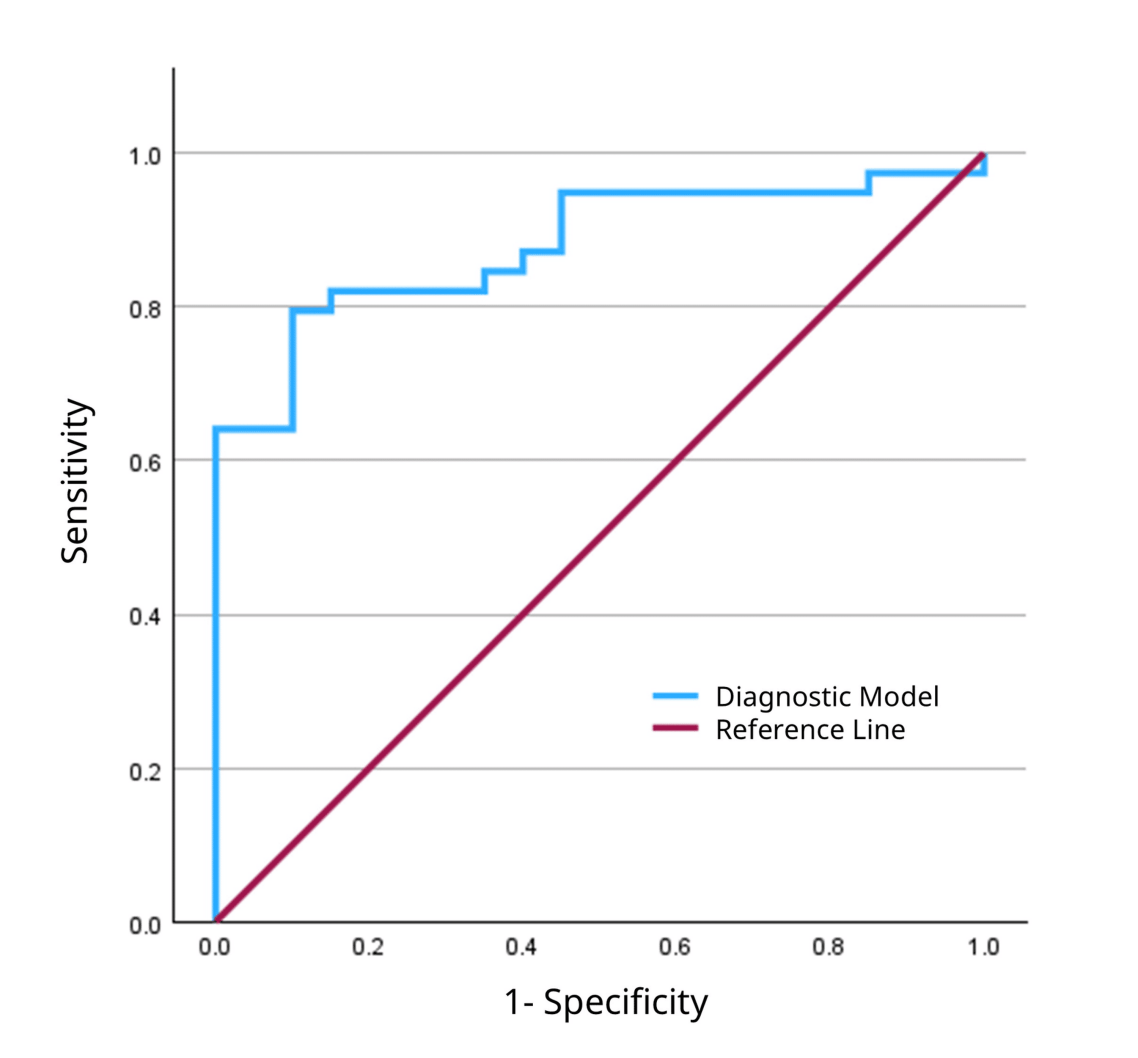
Receiver operating characteristic curve for the multivariable diagnostic model of hammer percussion. The model, which was adjusted for age, sex, body mass index, and osteoporosis, achieved an area under the curve of 0.879.

The overall discriminatory power of the adjusted models was excellent for manual percussion (AUC: 0.935; 95% CI: 0.876-0.993) (Figure 9) and good for hammer percussion (AUC: 0.879; 95% CI: 0.793-0.966) (Figure 10).

## Discussion

Summary and significance of the main results

This study demonstrated that objective, frequency-domain acoustic analysis of percussion sounds is a highly accurate screening tool for HF. Manual and hammer percussions exhibited strong diagnostic performance. A formal comparison using the DeLong test confirmed no statistically significant difference in AUC between the two methods. Crucially, the high sensitivity and extremely low negative likelihood ratio achieved with manual percussion highlight its exceptional utility as a rule-out tool. For example, a negative result can reduce a 40% pre-test probability of HF to just 2.3%. As demonstrated by the DCA, both methods offer a substantial net clinical benefit over default strategies across a wide range of threshold probabilities.

Comparison with previous methods

Our frequency-domain approach offers a marked improvement over existing methods. Conventional auscultatory percussion (PPPT) is limited by examiner subjectivity [10,11]. Prior attempts at objective acoustic diagnosis focused on sound amplitude (loudness), with a key study achieving a sensitivity of only 78% [12]. While direct comparisons must be made cautiously due to differences in study populations, methodologies, and reference standards, the high sensitivity achieved in our study (97.5%) contrasts favorably with the 78% sensitivity reported in the aforementioned study. This suggests that analyzing frequency, not just amplitude, may offer a substantial advantage in minimizing the risk of missed diagnoses, the most critical requirement for a screening tool.

Another portable screening modality is point-of-care ultrasound (POCUS), which also demonstrates high diagnostic accuracy for HF (e.g., a sensitivity of 83% and a specificity of 93% in one meta-analysis [17]). While POCUS is valuable, it typically requires more extensive training and more expensive equipment than the smartphone-based acoustic analysis presented here, potentially limiting its accessibility, particularly in resource-constrained settings [8].

Interpretation of results

The most scientifically notable finding is the dramatic difference in optimal diagnostic frequencies between the methods. Hard manual percussion using phalanges was most effective at high frequencies (2718.75 Hz), whereas soft hammer percussion using silicone rubber excelled at low frequencies (421.88 Hz). This is attributable to the physical properties of the impact. The Young modulus of bone (approximately 17 GPa) is orders of magnitude higher than that of silicone rubber (approximately 0.57-3.7 MPa) [18,19]. A hard, short-duration impact excites high-frequency components, whereas a soft, longer-duration impact concentrates energy at low frequencies [20].

These physical differences influence sound wave propagation. High-frequency waves travel most efficiently through dense cortical bone, acting as a direct test of continuity, as other pathways (cancellous bone or soft tissue) significantly attenuate these frequencies [21]. A fracture clearly disrupts this path, resulting in a robust signal. This robustness was confirmed by the consistently high performance of manual percussion across all subgroups and in the sensitivity analysis excluding patients with OA.

In contrast, low-frequency waves can travel through multiple pathways, reflecting complex acoustic transmission properties [22]. In subgroup analyses, hammer percussion trended toward higher accuracy in patients with osteoporosis than in those without. Although not statistically significant, this raises the hypothesis that low-frequency sounds may reflect overall bone integrity, a possibility warranting further investigation.

Although identifying a specific peak frequency (e.g., 2718.75 Hz) was crucial for demonstrating these physical principles, reliance on a single, narrow frequency bin (approximately 50 Hz wide) derived from this dataset may limit generalizability. Inter-patient variability or measurement conditions could slightly shift this optimal peak. Therefore, a more robust approach for future clinical application would involve transitioning from this "peak-based" metric to a "band-based" metric. This involves identifying broader, high-performance frequency bands (e.g., several hundred Hz wide) where diagnostic accuracy is consistently high. Validating such a band-based approach is an essential step toward developing a generalizable diagnostic algorithm, though it will require substantially larger datasets.

Clinical application and method selection

This method has high clinical applicability as a simple, non-invasive rule-out tool using common devices like smartphones, particularly valuable in settings with limited imaging access, such as nursing homes or developing countries [8].

Given the comparable diagnostic performance, the choice of method may depend on the user and context. For experienced clinicians, manual percussion requires no equipment, whereas for non-experts (e.g., paramedics and caregivers), hammer percussion might be technically easier to perform consistently. A larger-scale study is needed to definitively determine which method offers superior real-world diagnostic accuracy.

Traditional manual percussion presents two challenges: the technique of percussion itself and the subjective interpretation of the sound [23]. Our objective analysis resolves the challenge of subjective interpretation. Furthermore, this technology holds potential as an educational tool. By providing objective, quantifiable feedback, it could help standardize physical examination techniques among care providers.

Limitations

This study has some limitations that must be acknowledged.

Methodological and Statistical

The case-control design with a convenience‑sampled control group introduces spectrum and selection biases. More importantly, the modest overall sample and especially the small cell sizes within subgroups limit the precision and stability of all estimates. Although bootstrap internal validation was used to adjust for optimism, internal validation cannot fully mitigate overfitting in small samples; therefore, the reported AUC, sensitivity/specificity, and regression coefficients should be interpreted as preliminary and potentially optimistic. Consequently, generalizability to unselected, consecutive patients in emergency department/primary care settings, different operators and devices, and more diverse populations is inherently constrained and will require external validation. Finally, reliance on a single peak frequency identified within this dataset further narrows generalizability and underscores the need to validate broader, band‑based metrics in independent cohorts.

Procedural and Technical

Examiners were not blinded to the patient groups, introducing potential expectation bias, although this was mitigated by objective acoustic analysis. Inter-examiner variability remains a possibility, and formal inter-rater reliability was not assessed. The impulse selection process, while based on pre-defined criteria, was not fully automated (e.g., based on signal-to-noise ratio), leaving room for operator subjectivity. Formal acoustic calibration was not performed, and generalizability to other devices remains unverified.

Clinical Data Gaps

We did not collect data on fracture severity (e.g., Garden classification), bone mineral density (BMD), performance status, or Charlson comorbidity index, which may affect acoustic properties. Systematic assessment of pain and analgesic use during the procedure was lacking, which limits the full assessment of feasibility in acute settings. Crucially, the study design did not allow for the evaluation of performance in diagnostically challenging occult fractures (initially missed by radiography), as acoustic measurements were performed after the final diagnosis.

Generalizability

The cohort consisted primarily of older, non-obese Japanese patients. The reliability of this method in diverse populations, including those with severe obesity where subcutaneous fat could dampen the percussion impact, remains unverified.

Future directions

A large-scale, multicenter, prospective validation study including occult fractures is needed. Future work should focus on developing a more generalizable and fully automated algorithm. As discussed, this involves validating the transition from the narrow, peak-based metrics identified in this study to more robust, band-based metrics using large cohorts to ensure generalizability. Developing a smartphone application based on established methods for physiological sound analysis [24,25] could provide immediate diagnostic support at the point of care.

Furthermore, exploring optimized manual percussion techniques could enhance performance and usability. The "door-knock" technique (percussion with the proximal interphalangeal joint), which generates a hard, high-frequency signal and is intuitive for non-experts [26], may represent an ideal screening method. Finally, research linking low-frequency acoustic metrics to BMD data is warranted to explore its potential as a bone health assessment tool.

## Conclusions

This study demonstrated that objective, frequency-domain acoustic analysis of percussion sounds is a simple and effective screening tool for ruling out HFs. While manual and hammer percussions showed strong diagnostic accuracy, manual percussion achieved high sensitivity and a very low negative likelihood ratio, highlighting its particular utility as a rule-out tool. Scientifically, we found that the optimal diagnostic frequency depends on the physical properties of the percussion impact. This non-invasive, smartphone-based method holds substantial clinical potential; however, these promising results derived from a small, single-center case-control cohort require validation in larger, prospective studies. Future work should refine algorithms by moving from a narrow peak-based approach to more robust, band-based frequency metrics that aggregate performance across broader high-performing frequency ranges.
